# AI applications in HIV research: advances and future directions

**DOI:** 10.3389/fmicb.2025.1541942

**Published:** 2025-02-20

**Authors:** Ruyi Jin, Li Zhang

**Affiliations:** ^1^Department of Dermatology, The First Hospital of China Medical University, Shenyang, China; ^2^NHC Key Laboratory of Immunodermatology, China Medical University, Shenyang, China; ^3^Key Laboratory of Immunodermatology, China Medical University, Ministry of Education, Shenyang, China; ^4^National and Local Joint Engineering Research Center of Immunodermatological Theranostics, Shenyang, China

**Keywords:** HIV-human immunodeficiency virus, acquired immuno deficiency syndrome (AIDS), artificial intelligence - AI, machine learning, virology, deep learning

## Abstract

With the increasing application of artificial intelligence (AI) in medical research, studies on the human immunodeficiency virus type 1(HIV-1) and acquired immunodeficiency syndrome (AIDS) have become more in-depth. Integrating AI with technologies like single-cell sequencing enables precise biomarker identification and improved therapeutic targeting. This review aims to explore the advancements in AI technologies and their applications across various facets of HIV research, including viral mechanisms, diagnostic innovations, therapeutic strategies, and prevention efforts. Despite challenges like data limitations and model interpretability, AI holds significant potential in advancing HIV-1 management and contributing to global health goals.

## 1 Introduction

The human immunodeficiency virus type 1 (HIV-1) causes acquired immunodeficiency syndrome (AIDS). Since the World Health Organization (WHO) published guidelines in 2002 for treating HIV, advancements in antiretroviral therapy (ART) have notably increased both the life expectancy and quality of life for people living with HIV (PLWH) (World Health Organization, 2015). However, the world is still not on track to achieve the Sustainable Development Goal of ending AIDS as a public health threat by 2030 (Godfrey-Faussett et al., 2022). This highlights the urgent need to deepen our understanding of HIV and develop more effective treatment strategies and vaccines to address challenges posed by the virus’s variability and drug resistance.

**Figure 1:**
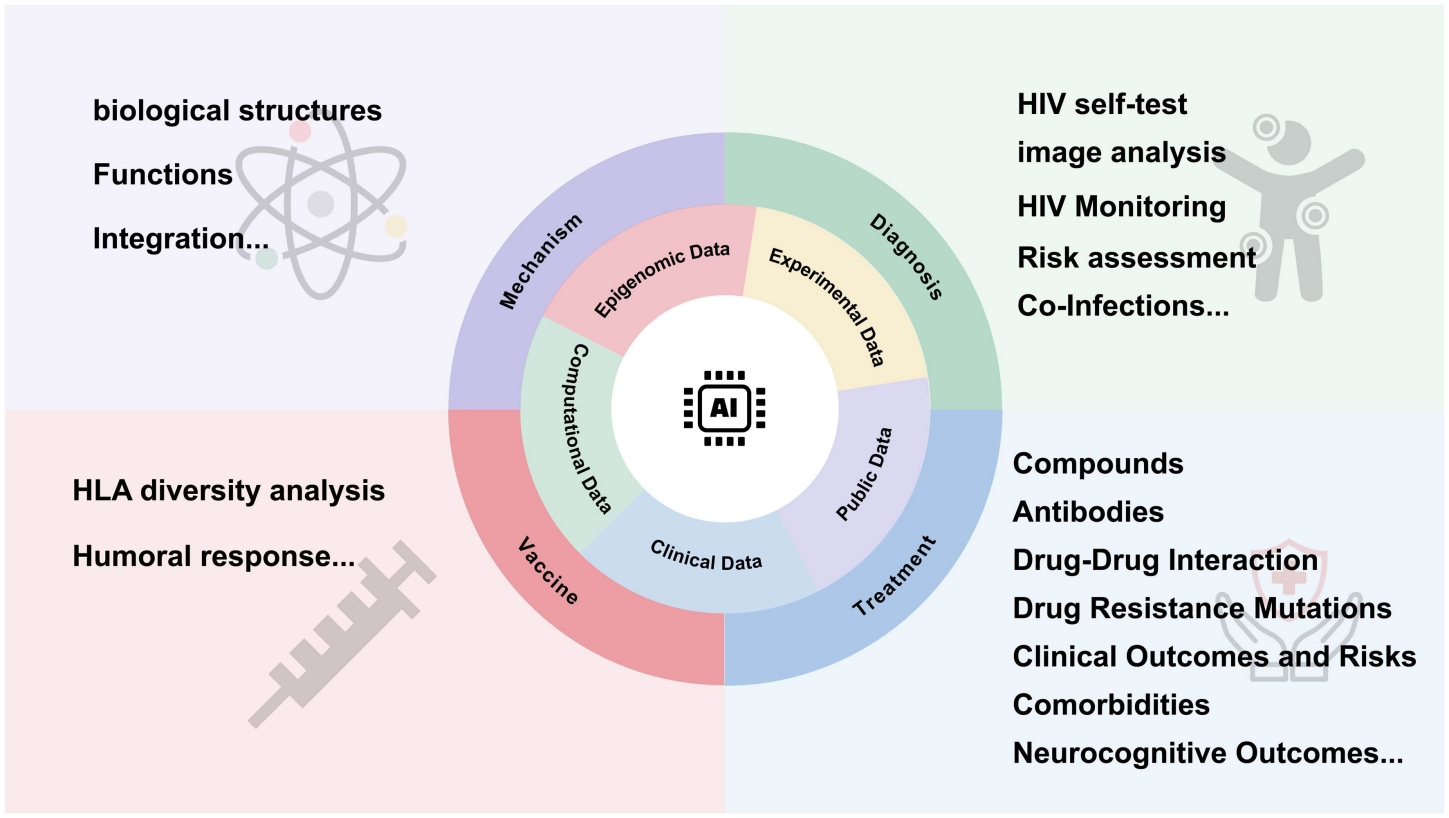
Overview of the key topics in this review.

Recent advancements in AI have opened new avenues in biomedical research. AI, using machine learning (ML) algorithms, can analyze vast biological datasets to enhance early detection, personalized treatment, and vaccine development (Wang et al., 2024; Hu et al., 2024; Yu et al., 2022; Holzinger et al., 2019). Machine learning algorithms particularly excel at building complex nonlinear models to link features with disease-related risk factors in large datasets, demonstrating both efficiency and accuracy (Li et al., 2024). As a subfield of AI, ML contains a diverse range of algorithm classes, linking to different learning tasks. These include supervised learning, unsupervised learning and reinforcement learning. Among these, the family of artificial neural networks have flexible structure, which enables adaptation to various situations in all three types of machine learning (Janiesch et al., 2021). Deep learning (DL) involves neural networks with multiple layers of computational neurons, enabling it to handle unstructured data like images (Petersen et al., 2022). The emergence of single-cell technologies has increased the availability of full-length paired B cell receptor (BCR) sequences. Consequently, combining immune repertoire sequencing with AI holds significant potential for improving the diagnosis and treatment of immune-related and infectious diseases (Irvine and Reddy, 2024; Figure 1).

Despite its potential, AI in HIV research faces challenges such as limited data access due to privacy concerns (You et al., 2020), ethical considerations (Gillette et al., 2023; Garett and Young, 2021), high variability of HIV and its complex immune evasion mechanisms, which complicate training and validation (Poonia and Al-Alshaikh, 2024; Vieira et al., 2017).

This review seeks to deliver a thorough overview of the latest advancements in AI within HIV research. It will also critically examine current challenges and propose future research directions.

## 2 Structural insights and integration mechanisms of HIV

### 2.1 HIV protein structure analysis

As a ML technique, support vector machine (SVM) is capable of executing both linear and nonlinear classification tasks (Pouyanfar et al., 2018). Another common statistical model, logistic regression (LR), can be used for both classification and regression problems (Sarker, 2021). In the quest to better understand HIV proteins, Mei’s study utilized Chou’s pseudo amino acid composition and increment of diversity as features, employing SVM, LR, and multilayer perceptron models to predict HIV-1 and HIV-2 proteins (Mei and Zhao, 2018). All three models demonstrated superior performance. Concurrently, the tomoDRGN (Powell and Davis, 2024) captures structural heterogeneity in cryo-electron tomography (cryo-ET) datasets through low-dimensional continuous representations, enabling the reconstruction of data-driven heterogeneous structures. This approach demonstrates exceptional performance in analyzing diverse datasets, including elucidating the organization of HIV capsid complexes within virus-like particles and resolving structural heterogeneity among ribosomes in cellular imaging.

### 2.2 Integration and tropism mechanisms of HIV-1

The integration of the HIV-1 genome into the human genome is a critical step in the viral infection and replication cycle influencing the persistence of infected cells (Maldarelli et al., 2014). Understanding this mechanism is essential for strategies to control the infection and its long-term effects.

DeepHINT (Hu et al., 2019), an attention-based deep learning framework, improves the prediction of HIV integration sites compared to traditional models. Unlike conventional approaches, DeepHINT automatically learned genomic context from DNA sequences, either independently or in conjunction with epigenetic information, providing both high accuracy and mechanistic insights.

HIV can infect cells at various stages of their development, ranging from monocytes to macrophages. A ML model has been employed to differentiate viral genomes in monocytes and T cells based on envelope sequences. This model identified five key features in the C2V3C3 region that significantly distinguish proviruses in monocytes: positions 297, 326, 335, 355, and 395 (Peng and Zhu, 2023). Additionally, XGBpred offer valuable insights into disease progression, underscoring the importance of computational methods in HIV research (Chen et al., 2019).

## 3 HIV diagnosis and co-infection

### 3.1 Advances in HIV diagnosis and monitoring tools

HIV self-test (HIVST) is an underutilized innovation in differentiated service delivery for HIV, despite its high sensitivity and specificity. Concerns about false negatives could delay antiretroviral therapy initiation and/or lead to inappropriate use of HIV pre-exposure prophylaxis (PrEP), which in turn increases the risk of HIV drug resistance (Gibas et al., 2019).

Two studies demonstrated the potential of AI in improving HIVST sensitivity through image analysis. Roche’s research (Roche et al., 2024) found that AI identified four HIV infections that were missed by both pharmacy providers and customers, highlighting its higher sensitivity in detecting faint lines on HIVST results. Similarly, Valérian Turbé’s deep learning algorithm (Turbé et al., 2021), trained on 11,374 images of rapid HIV tests from rural South Africa, achieved high sensitivity (97.8%), outperforming human interpretation in a pilot field study using a mobile app. Both studies demonstrated that the sensitivity of the AI algorithms surpassed that of traditional visual interpretation.

### 3.2 HIV monitoring and risk assessment

In HIV monitoring, real-time electronic adherence monitoring (EAM) combined with machine learning significantly improved the accuracy of predicting HIV viral load (Benitez et al., 2020). Additionally, researchers proposed a selective testing strategy to reduce testing frequency while effectively identifying high-risk patients. Another study (Balzer et al., 2020) used HIV testing data from Kenya and Uganda to develop a machine learning-based HIV risk score, which improved sensitivity in identifying high-risk individuals compared to traditional methods. Moreover, Bao et al. (2021) developed a model using demographic and sexual behavior data to predict the risk of HIV and sexually transmitted infections (STIs) among men who had sex with other men, yielding promising results.

### 3.3 Advances in managing HIV co-infections

HIV co-infections are common but crucial to study because they can accelerate disease progression and complicate treatment for both HIV and the co-infecting pathogen. Common HIV co-infections include viral hepatitis (HBV, HCV) (Li et al., 2024; Laguno et al., 2004; Wei et al., 2019; Sun et al., 2014; Muriuki et al., 2013; Wang et al., 2023), tuberculosis (Shen, 2024), and oncogenic viruses like HPV and HHV-8. These infections can worsen the overall health outcomes for HIV-positive individuals and increase risks of severe complications, such as liver disease and certain cancers. While ART has significantly improved HIV management, it cannot completely mitigate the additional risks posed by co-infections (Brites et al., 2021).

Firstly, HIV-1 and HCV share transmission routes, leading to a high risk of co-infection (Laguno et al., 2004). Researchers combined Naïve Bayes (NB) and SVM algorithms with two molecular fingerprints (MACCS and ECFP6) to develop 60 classification models, predicting compounds effective against 11 HIV-1 and 4 HCV targets. Over 20 potential multi-target inhibitors were identified, including seven HIV-1 and four HCV-approved drugs (Wei et al., 2019).

Co-infection with HIV and HBV is also prevalent due to similar transmission routes. Patients with HIV/HBV co-infection often experience faster disease progression, significantly increasing their risk of developing chronic liver disease, cirrhosis, end-stage liver disease, and hepatocellular carcinoma (Sun et al., 2014; Muriuki et al., 2013). Researchers applied ML to identify six novel compounds targeting both HIV-1 and HBV, demonstrating the potential for virtual screening in co-infection treatment (Wang et al., 2023).

In another study, Onywera et al. (2020) used LEfSe analysis to find that men co-infected with HIV and HPV showed a more diverse penile microbiota, although no significant association between HIV status and microbiota diversity was found.

Machine learning was also applied to predict early cervical cancer (CC) in women living with HIV, identifying key predictive factors, such as the duration of ART, WHO clinical stage, TPT status, viral load status, and family planning history. A Random Forest (RF) model suggested that ML can improve early detection rates for CC, reduce healthcare costs, and enhance preventive strategies (Namalinzi et al., 2024).

## 4 Innovations in treatment

### 4.1 Advancements in discovering HIV compounds and antibodies

Protease inhibitors remain an important component of ART for the treatment of HIV-1 infection (Mulato et al., 2024). Researchers have utilized AI techniques to screen datasets for potential HIV-1 protease ligands. These preliminary findings, validated through docking and molecular dynamics simulations, led to the discovery of a novel ligand outside known HIV-1 protease inhibitor classes (Arrigoni et al., 2023). Another study developed a gradient boosting model using a large dataset of HIV-1 protease inhibitors to predict ligand binding affinity. Enhanced by structural and potency data, it showed high accuracy. Shapley value analysis revealed that van der Waals interactions with key protein residues were critical to ligand potency (Leidner et al., 2019). Similarly, another study (Bitencourt-Ferreira et al., 2021) incorporated affinity data to further optimize small molecule drugs.

An approach integrated Long Short-Term Memory (LSTM) networks and variational autoencoders to accelerate HIV drug discovery to help identify candidate compounds and validate their interactions with HIV, offering a cost-effective drug development pathway (Kutsal et al., 2024).

Drug–drug interaction (DDI) prediction offers crucial insights for managing complex treatments effectively. Researchers have developed Deep-ARV, a tool designed to predict four categories of DDIs. To address the imbalance in DDI severity distribution, the model incorporates undersampling and ensemble learning techniques. This approach can help identify high-risk drug pairings, enhance the drug screening process, and support clinical drug development targeting DDI risks (Pham et al., 2024).

During the COVID-19 pandemic, researchers focused on identifying potential drugs targeting SARS-CoV-2, specifically the 3-chymotrypsin-like protease (3CLpro), an essential enzyme that plays a critical role in viral replication. Through computational screening methods, two compounds emerged as promising candidates against 3CLpro. These compounds are considered strong candidates for further development, offering a new direction for COVID-19 therapeutic strategies (Nand et al., 2020).

In antibody research, broadly neutralizing antibodies (bNAbs) against the HIV-1 envelope (Env) glycoprotein have shown promise for prevention and treatment. Using Rapid Automated Identification of bNAbs (RAIN) (Foglierini et al., 2024), researchers identified several bNAbs targeting the CD4 binding site of the HIV-1 Env glycoproteins. Structural analysis of bNAb4251, using cryo-electron microscopy, revealed that unconventional mutations are key to HIV-1 bNAbs’ function, offering new insights into HIV-1 immune evasion mechanisms.

### 4.2 Innovative predictive modeling and mutation analysis for addressing HIV drug resistance

To tackle HIV drug resistance, researchers have developed ML models to predict novel compounds that may inhibit HIV (Zorn et al., 2019). Due to HIV-1’s high sequence diversity and mutation rate, isolates often develop resistance to bNAbs. Traditionally, identifying resistant strains requires time-consuming *in vitro* assays. Reda Rawi used ML to predict HIV-1 resistance to 33 bNAbs, identifying key epitope features using gradient boosting machines. This in silico tool could streamline decision-making regarding antibody use and enable sequence-based monitoring of viral escape (Rawi et al., 2019). Steiner et al. (2020) evaluated three machine learning architectures—MLP, bidirectional recurrent neural network (Bi-RNN), and CNN—to predict resistance across 18 antiretroviral drugs. By combining sequence data with biological analysis, they enhanced the interpretation of drug resistance predictions.

Additionally, RF and SVM were employed to analyze resistance linked to 21 mutated residues in HIV target proteins. By using different kernel functions [linear, polynomial, and radial basis function (RBF)], these models offer insights into the impact of novel mutations on treatment efficacy (Cai et al., 2021).

Most antiretroviral drugs targeting HIV focus on reverse transcriptase (RT), yet HIV can develop drug resistance mutations (DRMs) under treatment pressure, limiting treatment options on a population level (Branda et al., 2024; Gupta et al., 2018). Traditionally, researchers have identified resistance mutations by comparing viral sequences from treated and untreated individuals (Willim et al., 2022), but this method tests mutations individually and fails to reveal interactions between them. In Luc Blassel’s study, ML methods were applied to analyze approximately 55,000 RT sequences from the UK. The analysis revealed six new mutations associated with resistance, providing candidates for further validation. They indicated that mutation interactions are primarily confined to the traditional paradigm, where primary DRMs confer resistance, while associated mutations modify resistance levels and/or offset the fitness costs linked to DRMs (Blassel et al., 2021).

### 4.3 Innovations in predictive modeling to address clinical outcomes and risks

Because of the challenges associated with gathering large cohort samples and comprehensive genetic data in clinical environments, data imbalance remains one of the significant challenges in applying ML to HIV research. To address this, researchers have combined ML with undersampling techniques, such as MAREV-1 and MAREV-2, aimed at identifying associations between Vif protein motifs and HIV clinical outcomes. These methods effectively identify genetic variants linked to HIV prognosis and mutations in accessory protein-coding regions, guiding new therapeutic strategies (Altamirano-Flores et al., 2023).

A meta-analysis of 24 studies involving 401,389 people living with HIV (PWH) evaluated machine learning models like random survival forests and SVM for predicting mortality risk. Machine learning showed strong potential for assessing long-term mortality risk, enhancing clinical decision-making (Li et al., 2024).

The persistent viral reservoir, particularly in CD4 T cells, remains a major obstacle to a cure. The complex interaction between the HIV reservoir and the host immune system made its characterization essential. A study (Semenova et al., 2024) identified significant correlations between immune cell populations and HIV reservoir size, including CD127 expression in CD4 T cells. Both intact and total proviral DNA levels showed a positive correlation with T cell activation and exhaustion, whereas ART duration and HIV-specific CD4 T cell responses were negatively correlated with intact provirus levels.

### 4.4 Machine learning insights into comorbidities and neurocognitive outcomes in HIV

Research involving PWH must consider comorbidities like cardiovascular disease, coinfections, and neurocognitive disorders, as these conditions often confound the primary effects of HIV (Long et al., 2016; Meyer-Rath et al., 2013; Calon et al., 2020).

Chronic HIV infection can lead to HIV-associated neurocognitive disorder (HAND). In a study conducted by Ogishi and Yotsuyanagi (2018), ML models identified key genetic features in the HIV env gene that predict HAND status. Specifically, three amino acid positions within the gp120 glycoprotein were predictive of HAND, with positions 291 and 315 being predictive of HIV-associated dementia (Holman and Gabuzda, 2012). These findings could guide the development of combination antiretroviral therapy (cART) regimens for HAND-associated quasispecies.

Peripheral nerve disorders (PNP) have been acknowledged for a long time as a characteristic of HIV-1 infection, becoming most evident as the disease progresses to AIDS (Kaku and Simpson, 2018). A study assessed PNP symptoms and signs through demographic, laboratory, and clinical variables. Using univariate and multivariate logistic regression combined with ML techniques, it identified key predictive factors for PNP. The most significant predictors included the duration of HIV-1 infection, peak plasma viral load, age, and low CD4+ T cell levels. These models improved classification performance and uncovered both known and novel factors, such as the duration of exposure to stavudine (Tu et al., 2021).

A subset of children with perinatal HIV (pHIV) faces persistent neurocognitive challenges (Ezeamama et al., 2016; Garvie et al., 2017; Bunupuradah et al., 2012). Recent studies have applied ML for neurocognitive development in pHIV children, demonstrating the feasibility of identifying those at risk for suboptimal outcomes. They also highlighted the interaction between HIV infection and mental health issues as early indicators of later neurocognitive challenges in pHIV children (Paul et al., 2020).

White matter changes in HIV-infected individuals can be quantified using brain-age gap (BAG) (Cole et al., 2017; Cole et al., 2018). Kalen J. Petersen used a Gaussian process regression model, trained on diffusion magnetic resonance imaging data from publicly available normative controls to assess BAG. Results indicated a significant interactive effect between BAG and detectable viral load (VL): PWH who had detectable VL exhibited an accumulation of +1.5 years in BAG per decade compared to HIV-negative controls (Petersen et al., 2022).

Studies have shown that the effects of smoking on DNA methylation in white blood cells (WBCs) can be detected using epigenome-wide association studies (EWAS) (You et al., 2020). Researchers used a DNA methylation-based ML approach to identify smoking-associated methylation sites predicting HIV prognosis and mortality. The chosen features successfully distinguished between favorable and poor HIV-related clinical outcomes in an independent sample. Additionally, the DNA methylation index derived from these CpGs was linked to mortality in the HIV-infected population. Notably, this study is the first to describe smoking-related DNA methylation associations in the HIV-infected population, demonstrating the utility of methylation-based machine learning in linking molecular information to clinical outcomes (Zhang et al., 2018).

## 5 Advancements in vaccine design

Over the past 15 years, numerous HIV-1 vaccine trials have failed to produce significant evidence of efficacy. Despite extensive efforts, the challenge of eliciting bNAbs has limited the success of these vaccine trials (Klasse and Moore, 2022).

Ed McGowan proposed a novel approach by analyzing HLA diversity and CD8 T cell immune responses, to identify key antigens (McGowan et al., 2021). Additionally, predicting viral strain sensitivity to monoclonal antibodies (mAbs) is critical for HIV vaccine and therapy development. Using amino acid sequence data and deep learning, researchers (Dănăilă and Buiu, 2022) have advanced the identification of potent antibody combinations by predicting HIV sensitivity to mAbs.

In a related effort, researchers developed a non-linear RF model using clinical and demographic data to predict the humoral response of PLWH to SARS-CoV-2 mRNA vaccines. This model suggests that additional booster doses may be necessary for PLWH, offering a tailored approach to vaccine strategies in clinical settings (Montesi et al., 2024).

## 6 Discussion

While AI is widely used and shows promising potential in HIV research, several challenges must be addressed.

AI models rely heavily on large, high-quality datasets to train algorithms effectively (Zou et al., 2023). Insufficient data can lead to incomplete training of AI models, which in turn affects the accuracy and reliability. Due to concerns over patient privacy, ethical issues, and the fragmentation of healthcare data, access to comprehensive HIV datasets remains limited especially across different populations and regions. In Emma Gillette’s study, participants identified accidental disclosure, stigma, and discrimination as significant risks of participating in research (Gillette et al., 2023). If participants lack confidence in researchers’ ability to protect data privacy and confidentiality, they may be discouraged from engaging in studies (Garett and Young, 2021). To ensure data security, one study implemented secure data governance rules and data encryption (Olatosi et al., 2019). Additionally, Zucchi proposed that, in some areas, adolescents need parental consent to participate in HIV research. Since it involved sensitive topics like sexual behavior, this requirement might violate their privacy and lead to embarrassment or even worse situations. Researchers suggested eliminating parental consent in ethical reviews to better respect, protect, and assist the participants (Zucchi et al., 2023).

Furthermore, HIV’s high genetic diversity and ability to evade the immune system present unique challenges (Dingens et al., 2017). HIV’s rapid mutation rate demands more sophisticated models to accurately predict viral behavior and other mechanisms, such as supporting long-term treatment and prevention strategies.

More importantly, the stigma surrounding AIDS (Guarnieri et al., 2024; Britton et al., 2024) places specific demands on AI technology. To be specific, false negatives may delay the initiation of ART (Gibas et al., 2019; Roche et al., 2024). Also, specificity is needed to avoid false positives that may cause psychological distress. Another important point is that AI models need to be accessible, facilitating testing in community and home settings to increase testing uptake and reduce stigma. In this way, the method can be considered highly practical by making testing more convenient and inclusive. It is advisable to conduct multicenter datasets and integrate a variety of ML algorithms for training. The goal is to create a more profound and powerful system to achieve a higher accuracy in analysis.

Collaboration among AI researchers, clinicians, and policymakers is of great importance. As mentioned above, removing parental consent can help not only protect adolescents but also enhance the size of datasets. Establishing ethical frameworks for data sharing, improving model transparency, and protecting data privacy are key to unlocking AI’s full potential in HIV research. With the advancement of AI models, personalized medicine and vaccines development could become a reality.

This review is a comprehensive study of AI applications in HIV research. It focuses on the integration of ML with other technologies, which previous literature has not explored enough. It aims to fill the gap in understanding how AI can enhance HIV researches and offer a new framework for future research. Also, it highlights the potential of AI to address current challenges in HIV management and contribute to global health goals.
